# On Modern Progress in Ophthalmic Medicine and Surgery

**Published:** 1894-04-21

**Authors:** Robert Brudenell Carter

**Affiliations:** Consulting Ophthalmic Surgeon to St. George's Hospital


### On Modern Progress in Ophthalmic Medicine and Surgery.

By Robert Brudenell Carter, F.R.C.S., Consulting Ophthalmic Surgeon to St. George's Hospital.

In order to make the incision required for his "modified linear extraction," Von Graefe found it necessary to abandon the triangular knife, and to adopt a narrow straight blade of uniform width for most of its length, but tapering equally to its point from back and edge. This knife, which has since been commonly called by his name, although it was really designed by Linnhardt, has continued, with slight modifications, to hold its ground, and is still in constant use. At first it was by no means without competitors, for Von Graefe's work gave a stimulus to the inventive energies of many other operators, and large numbers of people, who by some untoward accident found themselves in charge of ophthalmic hospitals, set themselves either to "invent" new instruments, or to "improve" the ordinary methods of using the old ones. With a few notable exceptions, the invention of a new instrument, or the origination of a new method, is a measure of the personal equation of the operator; that is to say, it is the expression of some particular incapacity on his part. He is awkward in the execution of a manœuvre which the old method or the old instrument requires, and he modifies one or the other in order to meet the necessities of his awkwardness. Many variations in the details of operating are of some value, in this way, to those by whom they are introduced; but do not possess the general value which would be necessary in order to secure their adoption by others. Most of the German suggestions of thirty or five-and-thirty years ago were of this personal character, and none which were so, as far as I am aware, have stood the test of experience. Some were almost comic. One learned person set himself to evacuate the lens through an incision straight across the cornea on its horizontal meridian, regardless of the inevitable alteration in its curvature and of the cicatrix in front of the pupil; and further, when this had been done, he enclosed the head of the hapless patient in a helmet of plaster of Paris bandage, which left only the mouth and nostrils free, and which was not disturbed or removed for a week. Another learned person devised a lance-knife, curved on the flat, to be used with its convex surface forwards, for the purpose of making an incision of sufficient length, which should yet lie wholly in the zone of tissue between the true cornea and the true sclera. The objection that such a knife could not receive a perfect edge was insufficient to damp the enthusiasm of the inventor. All sorts of hooks, single and double, were contrived for the purpose of dividing the capsule of the lens, and even cutting forceps were devised by which a piece of this capsule could be seized and withdrawn from the eye. The surgeon to a German ophthalmic hospital in those days appeared to feel that the introduction of some new method of cataract extraction was necessary to the maintenance of his reputation; and we in this country were inundated by German pamphlets which, for the most part, contained suggestions either opposed to the requirements of anatomy, or superfluous in the presence of operative skill. It was my melancholy duty to read the whole of them, and so to lay up a store of painful memories which are liable, even now, to return to me in the form of troubled dreams.

In the meanwhile, two improvements, of the greatest practical value, had been introduced in England, and served, more than anything else, to bring about a corresponding improvement in results. The late Mr. France, of Guy's Hospital, introduced the plan of steadying the eyeball by forceps applied to the conjunctiva, instead of by finger pressure; and the use of anæsthetics, which had long been avoided from fear of their tendency to produce vomiting, at length became general.

I have already described the way in which the fingers of the disengaged hand of the surgeon were employed, in flap-extraction, to raise the upper lid and to fix and steady the eyeball against the knife, and have also described the extreme nicety with which the pressure exercised by these fingers required to be graduated. At that time the lower lid was drawn down and fixed by an assistant. The plan adopted by Mr. France was to pinch up a vertical fold of the conjunctiva and sub-conjunctival tissue, immediately below the vertical meridian of the cornea. This plan entirely obviated the rolling inwards of the eye, or the exercise of any pressure upon it by which vitreous could be extruded; and it permitted the introduction of a wire speculum by which the lids were kept apart. The course of the knife could be seen from the first puncture to the completion of the incision; and the only compensating difficulty arose from the fact that the operators of that day had not sufficiently cultivated the art of using instruments in both hands at once, and of paying equal and undivided attention to them. Until this art had been acquired there was some liability to attend only to the knife, and unconsciously to exercise pressure or traction with the forceps. This difficulty once overcome, there was an end of the complications which had previously so often been produced by a faulty direction of the incision.

The next great step was the introduction of anæsthetics. Prior to their employment vitreous was liable to be expelled, not only by the unskilful finger pressure of the operator, but also by the contractions of the ocular muscles of the patient. These muscles endeavoured instinctively to remove the eye from pain, and especially the superior rectus would endeavour to roll it up beneath the shelter of the upper lid. Such efforts were little, if at all, under voluntary control, and they exposed the hyaloid membrane to considerable risk of rupture, as a result of which not only would vitreous escape, but the lens would sometimes fall back deeply into the vitreous chamber, and be extracted, if at all, only with considerable difficulty, and with very little prospect of benefit. The injurious muscular contractions were even more likely to arise under forceps fixation than under finger pressure, the grip of the forceps being an additional exciting cause. As long as flap-extraction was practised, the large size of the corneal wound, and the position of the iris with regard to it, rendered even a very small amount of vomiting highly dangerous, because it could hardly fail to force the iris between the edges of the wound, and so to produce at the best delayed healing, with alteration of the pupillary outline and of the corneal curvature. But, when the external wound was placed in a less mobile region, and when the piece of iris immediately in front of it had been excised, a moderate amount of vomiting entailed comparatively little danger, and surgeons gradually began to employ chloroform in the majority of their cases of extraction, so as to avail themselves of the muscular quiescence which it secured, and of the deliberateness of procedure which it rendered possible.

